# Crystal structures of 2,3-bis­(thio­phen-2-yl)pyrido[2,3-*b*]pyrazine and 7-bromo-2,3-bis­(thio­phen-2-yl)pyrido[2,3-*b*]pyrazine

**DOI:** 10.1107/S2056989018016882

**Published:** 2019-01-01

**Authors:** Rafal Popek, Guy Crundwell

**Affiliations:** a Department of Chemistry & Biochemistry, Central Connecticut State University, New Britain, CT 06053, USA

**Keywords:** crystal structure, pyrido[2,3-*b*]pyrazines, offset π-π inter­actions, hydrogen bonding

## Abstract

The crystal structures of 2,3-bis­(thio­phen-2-yl)pyrido[2,3-*b*]pyrazine, C_15_H_9_N_3_S_2_ (**1**), and 7-bromo-2,3-bis­(thio­phen-2-yl)pyrido[2,3-*b*]pyrazine, C_15_H_8_BrN_3_S_2_ (**2**), are discussed. Both mol­ecules crystallize in space group *P*2_1_/*c*. In **1**, the thienyl rings are inclined to the mean plane of the pyrido­pyrazine moiety by 6.16 (7) and 86.66 (8)°, where as in **2** the corresponding dihedral angles are 33.29 (11) and 19.84 (9)°. The pyrido­pyrazine moiety is relatively planar in **1** with the two rings being inclined to each other by 1.33 (7)°. In **2**, however, the pyrido­pyrazine moiety is buckled with the corresponding dihedral angle being larger at 8.78 (10)°. In the crystal of **1**, the packing creates inter­secting bilayers; the layering results from the pyrido­pyrazine moieties being engaged in offset π-stacking, where the inter­planar distance is 3.431 (9) Å with an offset 1.14 Å. In the crystal of **2**, the mol­ecules pack head-to-head and are linked by a series of C—H⋯Br and C—H⋯N inter­molecular inter­actions, forming layers parallel to the *ab* plane.

## Abstract 2

In 2,3-di(thio­phen-2-yl)pyrido[2,3-*b*]pyrazine the thienyl rings are inclined to the mean plane of the pyrido­pyrazine moiety by 6.16 (7) and 86.66 (8)°, where as in the bromo derivative, 7-bromo-2,3-bis­(thio­phen-2-yl)pyrido[2,3-*b*] pyrazine, the corresponding dihedral angles are 33.29 (11) and 19.84 (9)°.

## Chemical context   

Nitro­gen-containing heterocyclic aryl substituents at the 2- and 2,3- positions on quinoxalines have been shown repeatedly to engage in bidentate behavior in binding metals, utilizing the quinoxaline nitro­gen atom. For example, 2-(2-pyrid­yl)quinoxaline has shown bidentate behavior with a variety of metals; focusing on silver, specifically, it can form 1:1 complexes assembling in one-dimensional chains (Shanmuga Sundara Raj *et al.*, 1999 ▸) or form 2:1 mononuclear complexes (Bi *et al.*, 2009 ▸) to cite just a few. With that bidentate behavior in mind, we aimed to test the bonding capabilities of thienyl sulfur atoms at the 2-, and 2,3- positions on mono- and di-thienylquinoxalines. Thienyl-substituted quinoxalines have been shown to form bis-complexes with silver(I) (Crundwell *et al.*, 2014 ▸; Crundwell, 2013 ▸); however, so far we have not seen (N,S) bidentate behavior from the nitro­gen on the quinoxaline and sulfur on the thienyl ring with a metal.

Monothienyl and/or 2,3-dithienyl-substituted pyrido[2,3-*b*]pyrazines are inter­esting ligands related to their quinoxaline analogs since they have an additional heterocyclic nitro­gen atom. This could potentially create novel silver(I) frameworks and allow insight into the preference of silver when it binds to the heterocycles in these ligands. To date, little work has been done with monothienylpyrido[2,3-*b*]pyrazines or 2,3-dithien­yl­pyrido[2,3-*b*]pyrazines. The crystal structure of 3-(2-thien­yl)pyrido[2,3-*b*]pyrazine has been determined (Lassagne *et al.*, 2015 ▸). A few other 2,3-di­aryl­pyrido[2,3-*b*]pyrazines and their subsequent metal complexes have been characterized through diffraction studies. The crystal structure of 2,3-di(1*H*-2-pyrrol­yl)pyrido[2,3-*b*]pyrazine, which is a colormetric ion sensor, has been determined as well as a nickel(II) complex in which two ligands bind to the nickel via the outermost nitro­gen atom on the pyrido­pyrazine moiety (Ghosh *et al.*, 2006 ▸). Rhenium(I) complexes with the generic formula [ReBr(CO)_3_(*L*)] have been synthesized with a few 2,3-di­aryl­pyrido[2,3-*b*]pyrazines (Yeo *et al.*, 2010 ▸). These complexes are inter­esting because they utilize both nitro­gen atoms on the same side of the pyrido­pyrazine moiety to bind the metal.
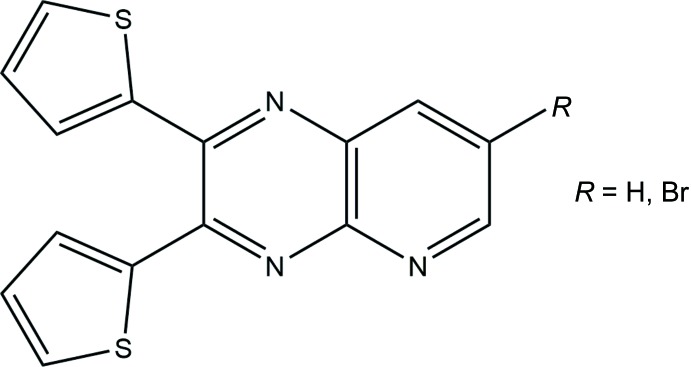


## Structural details   

**Figure 1:**
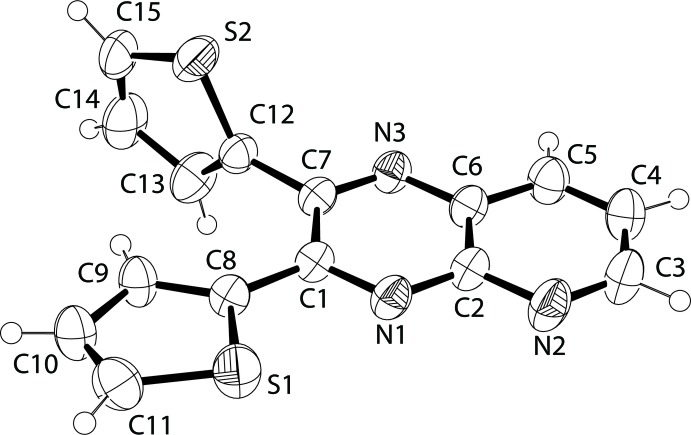
A view of the mol­ecular structure of compound **1**, with the atom labeling and displacement ellipsoids drawn at the 50% probability level.

The mol­ecular structure of compound **1** is shown in Fig. 1 ▸. One of the two thienyl rings (C8–C11/S1) is nearly coplanar with the pyrido­pyrazine ring [the dihedral angle being 6.16 (7)°], therefore making most of the mol­ecule appear flat. The r.m.s. deviation for all non-hydrogen atoms in the pyrido­pyrazine moiety and the nearly coplanar thio­phene ring (C8–C11/S1) is only 0.0123 (16) Å. The mean plane of the other thienyl ring (C12–C15/S2) is nearly perpendicular to the plane created by the rest of the mol­ecule, forming an angle of 86.67 (4)°. Finally, although unsubstituted thienyl ring-flip disorders are common on unsubstituted 2- or 3-thienyl rings (Crundwell *et al.*, 2003 ▸), there was not enough evidence of such a disorder to include it in the refinement model for **1**.

**Figure 2:**
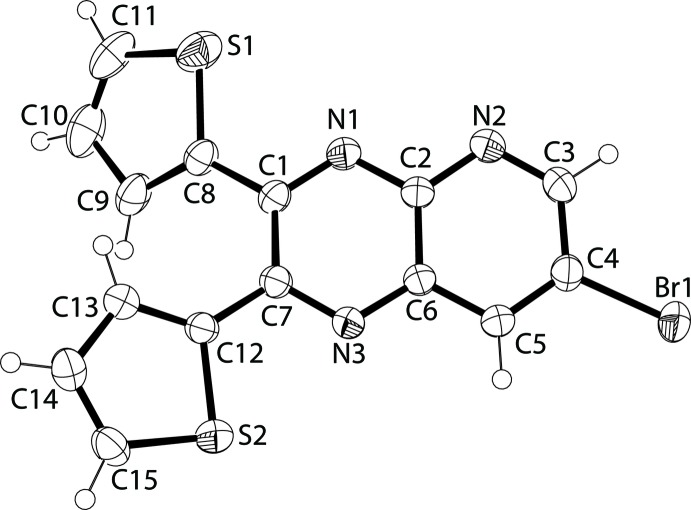
A view of the mol­ecular structure of compound **2**, with the atom labeling and displacement ellipsoids drawn at the 50% probability level.

The mol­ecular structure of compound **2** is shown in Fig. 2 ▸. This bromo derivative is less planar than the unbrominated compound **1**. The r.m.s. atomic displacement for the non-hydrogen atoms in the pyrido­pyrazine ring is 0.104 (2) Å. The mean planes of the thienyl rings (C8–C11/S1 and C12–C15/S2) form angles of 33.29 (11) and 19.84 (9)°, respectively, with the mean plane of the pyrido­pyrazine moiety. The later is buckled with the pyrazine and pyridine rings being inclined to each other by 8.78 (10)°, compared to only 1.33 (7) ° in **1**.

All bond lengths and angles in both compounds **1** and **2** are within expected values and close to those reported for similar compounds (see *Database survey*).

## Supra­molecular Features   

**Figure 3:**
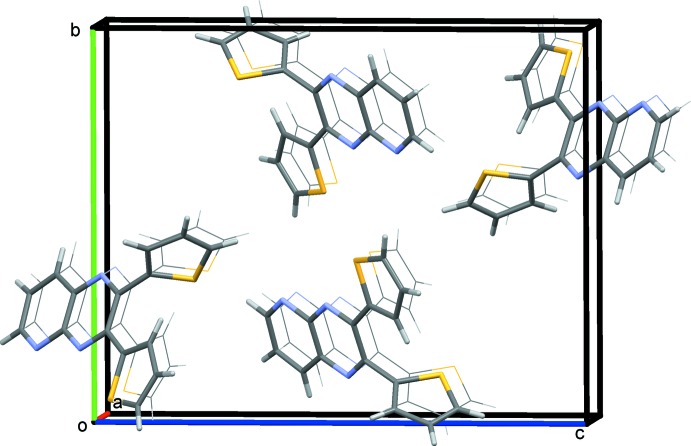
A view along the *a* axis of the crystal packing of compound **1**. Extra mol­ecules were added to illustrate the stacking that occurs in planes.

**Figure 4:**
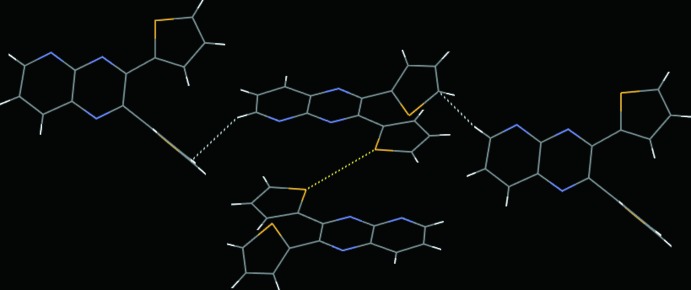
Inter­molecular inter­actions in the crystal of **1**. The S⋯S inter­actions are shown as dotted yellow lines. The C—H⋯π (thienyl ring) inter­actions are shown in white.

In the crystal of **1**, the packing can be described as a series of bilayers (Fig. 3 ▸). Using *Mercury* software (Macrae *et al.*, 2008 ▸) for the analysis, in can be seen that the mol­ecules lie in planes with an offset π-stacking distance of 3.431 (9) Å, measured between the planar thienyl ring in one mol­ecule and a portion of the pyrido­pyrazine ring system of a neighboring mol­ecule. There are two other types of very weak inter­molecular inter­actions in the crystal. The thienyl-ring sulfur atom S1 points directly at a neighboring inversion-related co-planar thienyl-ring sulfur atom at a distance of 3.570 (8) Å, roughly comparable to the sum of the van der Waals radii (3.8 Å). In addition, the pyrido­pyrazine hydrogen atom H3 is in a position to inter­act with the *sp* ^2^ carbon atom C15^i^ on the tilted thienyl ring (C12–C15/S2) at (i) *x* + 1, −*y* + 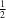, *z* − 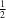, at a distance of 2.870 (8) Å and forming an angle C3—H3⋯C15^i^ of 152.37 (8)°. These inter­actions are shown as colored dotted lines in Fig. 4 ▸.

**Figure 5:**
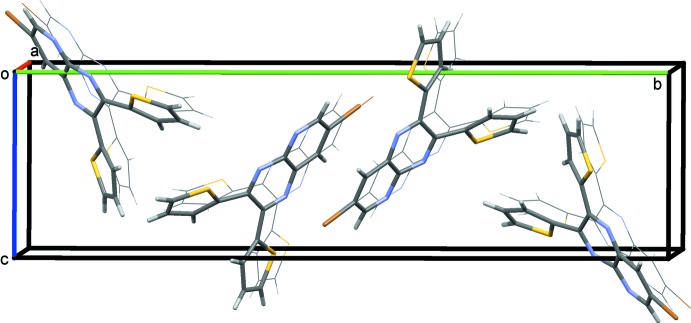
A view along the *a* axis of the crystal packing of compound **2**. Extra mol­ecules were added to illustrate the stacking that occurs in planes.

**Figure 6:**
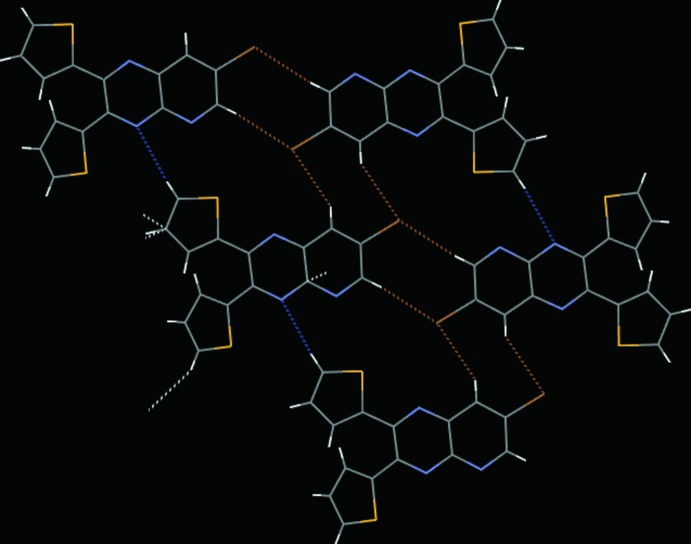
Inter­molecular inter­actions in the crystal of **2**, highlighting the two-dimensional network of C—H⋯Br (brown dotted lines) and C—H⋯N (blue dotted lines) inter­actions that lie in the same plane. The dangling contacts on the thienyl rings, indicating C—H⋯π (thienyl ring) and C—H⋯π (pyrido­pyrazine) inter­actions, are shown with white dotted lines.

In the crystal of the brominated derivative **2**, mol­ecules pack through a number of inter­molecular inter­actions (Fig. 5 ▸, Table 1 ▸). Several inter­actions between the bromine atoms and neighboring hydrogens create a head-to-head, sheet-like structure (Fig. 6 ▸). Bromine atoms form C—H⋯Br contacts at distances of 3.005 and 3.049 Å with the hydrogen atoms on C5 and C3, respectively. Within the same plane there are also inter­actions between the pyrido­pyrazine nitro­gen atom, N1, and adjacent thienyl-ring hydrogen atoms on C15 at 2.645 Å. Finally, two types of inter­actions that connect mol­ecules between planes are also present. A thienyl-ring hydrogen (on C11) is in contact with an *sp* ^2^ carbon (C14) in another layer at 2.775 Å and the π-system of the C12–C15/S2 thienyl ring is stacked over a neighboring pyrido­pyrazine moiety at 3.394 (9) Å. These inter­actions are shown as colored dotted lines in Fig. 6 ▸.

## Database Survey   

A search of the CSD (Version 5.39, August 2018 update; Groom *et al.*, 2016 ▸) revealed the crystal structures of two other aryl­pyrido[2,3-*b*]pyrazines, in addition to those already mentioned in the *Chemical context* section. In 7-bromo-3-[4-(piperidin-1-yl)phen­yl]pyrido[2,3-*b*]pyrazine, the brominated pyrido­pyrazine ring remains coplanar with its aryl substituent (CSD refcode MUPVOK; Kekesi *et al.*, 2014 ▸). The same result is not found for 2,3-bis­(5-bromo-1*H*-indol-3-yl)-7-chloro­pyrido[2,3-*b*]pyrazine acetone monosolvate (JUGCOF; Manivannan *et al.*, 2015 ▸), whose conformation resembles that of compound **2**, with both substituents being inclined to the mean plane of the pyrido­pyrazine ring.

Pyrido[2,3-*b*]pyrazines without halogenated pyrido­pyrazine rings are prevalent in the literature. Examples include: 2-(4-fluoro­phen­yl)-3-(pyridin-4-yl)pyrido[2,3-*b*]pyrazine (BUD­YAB; Koch *et al.*, 2009*a* ▸), 4-[3-(4-fluoro­phen­yl)pyrido[2,3-*b*]pyrazin-2-yl]-*N*- iso­propyl­pyridin-2-amine (BUFBAG; Koch *et al.*, 2009*c* ▸), 3-(4-fluoro­phen­yl)-2-(pyridin-4-yl)pyrido[2,3-*b*]pyrazine (PUFNUA; Koch *et al.*, 2009*b* ▸), 4,4′-pyrido[2,3-*b*]pyrazine-2,3-diylbis(*N*,*N*-di­phenyl­aniline) (WUDQAO, WUDQAO01; Xu *et al.*, 2015 ▸) and 4′,4′′-(pyrido[2,3-*b*]pyrazine-2,3-di­yl)bis­[(1,1′-biphen­yl)-4-carbo­nitrile]­chloro­form monosolvate (YEMQUF; Gupta *et al.*, 2018 ▸). In all of these structures, both substituents are inclined to the mean plane of the pyrido­pyrazine ring, similar to the situation in compound **2**.

## Synthesis and crystallization   

All reagents were purchased from Sigma Aldrich and used without purification. Both mol­ecules were synthesized by reacting equimolar amounts of the corresponding 2,3-di­amino­pyridines with 2,2′-thenil in refluxing glacial acetic acid.

**2,3-Bis(thio­phen-2-yl)pyrido[2,3-** ***b*** **]pyrazine (1)**: To a 250 ml round-bottom flask equipped with a magnetic stir bar were added 0.570 g of 2,3-di­amino­pyridine (5.23 mmol), 1.160 g of 2,2′-thenil (5.23 mmol), and 150 ml of glacial acetic acid. The solution was stirred, heated to boiling, and refluxed for 3 h. The resulting yellowish-brown solution was poured into a 250 ml beaker filled with ice, neutralized with sodium hydroxide, and isolated using vacuum filtration. A rough yield of the yellow–brown solid was 1.332 g (77%). The product was purified via column chromatography (SiO_2_, 80% EtOAc/20% hexane, *R*_f_ = 0.75) to yield 1.010 g of compound **1** (m.p. 451 K). ATR–IR (cm^−1^) 3101, 1541, 1453, 1409, 1359, 1257, 1092; ^1^H NMR (300 MHz, CDCl_3_): δ 9.50 (*d*, 1H), 8.79 (*d*, 1H), 7.90 (*dd*, 1H), 7.59 (*m*, 2H), 7.38 (*dd*, 2H), 7.10 (*m*, 2H); ^13^C NMR (300 MHz, CDCl_3_): δ 154.28, 149.68, 149.31, 147.68, 141.17, 140.56, 137.63, 135.62, 130.60, 130.46, 129.96, 129.45, 127.70, 127.64, 125.12. Yellow plate-like crystals of **1** were obtained by slow evaporation of a solution in an equal volume mixture of toluene and ethanol.

**7-Bromo-2,3-bis­(thio­phen-2-yl)pyrido[2,3-** ***b*** **]pyrazine (2)**: The above method was used for the brominated derivative by using 5-bromo-2,3-di­amino­pryidine as the starting di­amine (m.p. 445 K); ATR–IR (cm^−1^) 3099, 1539, 1427, 1410, 1331, 1311, 1237, 1172, 1072; ^1^H NMR (300 MHz, CDCl_3_): δ 9.10 (*d*, 1H), 8.58 (*d*, 1H), 7.59 (*m*, 2H), 7.46 (*m*, 2H), 7.10 (*m*, 2H); ^13^C NMR (300 MHz, CDCl_3_): δ 155.14, 149.73, 148.44, 148.03, 140.83, 140.36, 138.89, 135.82, 130.88, 130.66, 130.33, 130.07, 127.78, 127.72, 120.81. Yellow plate-like crystals of **2** were obtained by slow evaporation of a solution in an equal volume mixture of toluene and ethanol. ^1^H, FTIR, and COSY NMR spectra for **2** are given in the supporting information.

## Refinement   

Crystal data, data collection and structure refinement details are summarized in Table 2 ▸. All the hydrogen atoms were constrained at ideal positions and refined using a riding model: C—H = 0.93Å with *U*_iso_(H) = 1.2*U*_eq_(C). In both compounds, some reflections were omitted because they were either partially obstructed by the beam stop or they had an Error/e.s.d. ratio higher than 5.00 where Error = Σ(*D*)(*wD* ^2^/<*wD* ^2^)^0.5^, *D* being *F*_c_^2^ − *F*_o_^2^.

## Supplementary Material

Crystal structure: contains datablock(s) 1, Global, 2. DOI: 10.1107/S2056989018016882/xi2011sup1.cif

Structure factors: contains datablock(s) 1. DOI: 10.1107/S2056989018016882/xi20111sup2.hkl

Supporting information file. DOI: 10.1107/S2056989018016882/xi20111sup4.cml

Structure factors: contains datablock(s) 2. DOI: 10.1107/S2056989018016882/xi20112sup3.hkl

Supporting information file. DOI: 10.1107/S2056989018016882/xi20112sup5.cml

NMRs and FTIRs of 1 and 2. DOI: 10.1107/S2056989018016882/xi2011sup6.pdf

CCDC references: 1881685, 1881684

Additional supporting information:  crystallographic information; 3D view; checkCIF report

## Figures and Tables

**Table 1:** Hydrogen-bond geometry (Å, °) for **2** [Chem scheme1]

*D*—H⋯*A*	*D*—H	H⋯*A*	*D*⋯*A*	*D*—H⋯*A*
C3—H3⋯Br1^i^	0.93	3.01	3.836 (1)	150
C5—H5⋯Br1^ii^	0.93	3.05	3.851 (1)	145
C15—H15⋯N1^iii^	0.93	2.65	3.572 (1)	175
C11—H11⋯C14^iv^	0.93	2.78	3.637 (1)	155

Symmetry codes: (i) 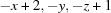; (ii) 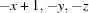; (iii) 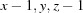; (iv) 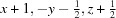.

**Table 2:** Experimental details

	**1**	**2**
Crystal data		
Chemical formula	C_15_H_9_N_3_S_2_	C_15_H_8_BrN_3_S_2_
*M*_r_	295.37	374.27
Crystal system, space group	Monoclinic, *P*2_1_/*c*	Monoclinic, *P*2_1_/*c*
Temperature (K)	293	293
*a*, *b*, *c* (Å)	5.25147 (12), 14.1093 (3), 17.7690 (3)	5.8336 (2), 29.4731 (10), 8.3160 (3)
β (°)	92.0296 (18)	95.466 (3)
*V* (Å^3^)	1315.76 (4)	1423.30 (9)
*Z*	4	4
Radiation type	Mo *K*α	Mo *K*α
μ (mm^−1^)	0.40	3.18
Crystal size (mm)	0.43 × 0.33 × 0.21	0.33 × 0.24 × 0.22

Data collection		
Diffractometer	Rigaku Xcalibur Sapphire3	Rigaku Xcalibur Sapphire3
Absorption correction	Multi-scan (*CrysAlis PRO*; Rigaku OD, 2018 ▸)	Multi-scan (*CrysAlis PRO*; Rigaku OD, 2018 ▸)
*T*_min_, *T*_max_	0.948, 1.000	0.455, 1.000
No. of measured, independent and observed [*I* > 2σ(*I*)] reflections	19251, 4763, 3491	34519, 5255, 4166
*R*_int_	0.021	0.034
(sin θ/λ)_max_ (Å^−1^)	0.773	0.785

Refinement		
*R*[*F* ^2^ > 2σ(*F* ^2^)], *wR*(*F* ^2^), *S*	0.048, 0.148, 1.01	0.043, 0.110, 1.08
No. of reflections	4763	5255
No. of parameters	181	190
H-atom treatment	H-atom parameters constrained	H-atom parameters constrained
Δρ_max_, Δρ_min_ (e Å^−3^)	0.44, −0.40	0.63, −0.67

Computer programs: *CrysAlis PRO* (Rigaku OD, 2018 ▸), *SHELXS97* and *SHELXL97* (Sheldrick, 2008 ▸), *ORTEP-3 for Windows* (Farrugia, 2012 ▸), *Mercury* (Macrae *et al.*, 2008 ▸) and *OLEX2* (Dolomanov *et al.*, 2009 ▸).

## supplementary crystallographic information

### 2,3-Bis(thiophen-2-yl)pyrido[2,3-b]pyrazine (1) Crystal data

**Table:**

C_15_H_9_N_3_S_2_	*D*_x_ = 1.491 Mg m^−^^3^
*M**_r_* = 295.37	Melting point: 451 K
Monoclinic, *P*2_1_/*c*	Mo *K*α radiation, λ = 0.71073 Å
*a* = 5.25147 (12) Å	Cell parameters from 6256 reflections
*b* = 14.1093 (3) Å	θ = 4.5–32.1°
*c* = 17.7690 (3) Å	µ = 0.40 mm^−^^1^
β = 92.0296 (18)°	*T* = 293 K
*V* = 1315.76 (4) Å^3^	Plate, yellow
*Z* = 4	0.43 × 0.33 × 0.21 mm
*F*(000) = 608	

### 2,3-Bis(thiophen-2-yl)pyrido[2,3-b]pyrazine (1) Data collection

**Table:**

Rigaku Xcalibur Sapphire3 diffractometer	4763 independent reflections
Radiation source: Enhance (Mo) X-ray Source	3491 reflections with *I* > 2σ(*I*)
Graphite monochromator	*R*_int_ = 0.021
Detector resolution: 16.1790 pixels mm^-1^	θ_max_ = 33.3°, θ_min_ = 4.1°
ω scans	*h* = −8→6
Absorption correction: multi-scan (CrysAlis PRO; Rigaku OD, 2018)	*k* = −21→21
*T*_min_ = 0.948, *T*_max_ = 1.000	*l* = −27→26
19251 measured reflections	

### 2,3-Bis(thiophen-2-yl)pyrido[2,3-b]pyrazine (1) Refinement

**Table:**

Refinement on *F*^2^	0 restraints
Least-squares matrix: full	H-atom parameters constrained
*R*[*F*^2^ > 2σ(*F*^2^)] = 0.048	*w* = 1/[σ^2^(*F*_o_^2^) + (0.0773*P*)^2^ + 0.306*P*] where *P* = (*F*_o_^2^ + 2*F*_c_^2^)/3
*wR*(*F*^2^) = 0.148	(Δ/σ)_max_ < 0.001
*S* = 1.01	Δρ_max_ = 0.44 e Å^−^^3^
4763 reflections	Δρ_min_ = −0.40 e Å^−^^3^
181 parameters	

### 2,3-Bis(thiophen-2-yl)pyrido[2,3-b]pyrazine (1) Special details

**Table:**

Geometry. All esds (except the esd in the dihedral angle between two l.s. planes) are estimated using the full covariance matrix. The cell esds are taken into account individually in the estimation of esds in distances, angles and torsion angles; correlations between esds in cell parameters are only used when they are defined by crystal symmetry. An approximate (isotropic) treatment of cell esds is used for estimating esds involving l.s. planes.
Refinement. Refinement of F^2^ against ALL reflections. The weighted R-factor wR and goodness of fit S are based on F^2^, conventional R-factors R are based on F, with F set to zero for negative F^2^. The threshold expression of F^2^ > 2sigma(F^2^) is used only for calculating R-factors(gt) etc. and is not relevant to the choice of reflections for refinement. R-factors based on F^2^ are statistically about twice as large as those based on F, and R- factors based on ALL data will be even larger.

### 2,3-Bis(thiophen-2-yl)pyrido[2,3-b]pyrazine (1) Fractional atomic coordinates and isotropic or equivalent isotropic displacement parameters (Å^2^)

**Table:**

	*x*	*y*	*z*	*U*_iso_*/*U*_eq_	
S1	0.29005 (9)	0.07264 (3)	0.05184 (3)	0.05601 (15)	
S2	0.68406 (10)	0.36939 (5)	0.21271 (3)	0.0735 (2)	
N3	0.8135 (2)	0.37660 (8)	0.02149 (7)	0.0390 (3)	
N1	0.6466 (3)	0.19393 (8)	−0.01845 (7)	0.0407 (3)	
C1	0.5489 (3)	0.23948 (9)	0.03890 (7)	0.0358 (3)	
C7	0.6384 (3)	0.33311 (9)	0.05955 (7)	0.0356 (3)	
C8	0.3498 (3)	0.18831 (9)	0.07804 (8)	0.0371 (3)	
C12	0.5459 (3)	0.38619 (10)	0.12539 (8)	0.0401 (3)	
C2	0.8310 (3)	0.23713 (10)	−0.05676 (8)	0.0387 (3)	
C6	0.9139 (3)	0.32947 (10)	−0.03738 (8)	0.0381 (3)	
N2	0.9315 (3)	0.18788 (10)	−0.11470 (8)	0.0531 (3)	
C11	0.0556 (4)	0.06156 (12)	0.11368 (11)	0.0539 (4)	
H11	−0.0380	0.0063	0.1198	0.065*	
C9	0.1873 (3)	0.21596 (11)	0.13489 (9)	0.0439 (3)	
H9	0.1894	0.2753	0.1576	0.053*	
C10	0.0188 (3)	0.14123 (12)	0.15320 (10)	0.0523 (4)	
H10	−0.1050	0.1470	0.1891	0.063*	
C5	1.1070 (3)	0.37201 (11)	−0.07888 (10)	0.0483 (3)	
H5	1.1647	0.4329	−0.0676	0.058*	
C4	1.2062 (4)	0.32159 (14)	−0.13583 (10)	0.0532 (4)	
H4	1.3348	0.3471	−0.1642	0.064*	
C3	1.1122 (4)	0.23039 (14)	−0.15125 (10)	0.0567 (4)	
H3	1.1835	0.1973	−0.1906	0.068*	
C13	0.3588 (4)	0.45253 (13)	0.12665 (11)	0.0561 (4)	
H13	0.2629	0.4718	0.0844	0.067*	
C15	0.4890 (4)	0.45263 (18)	0.25111 (12)	0.0691 (6)	
H15	0.4964	0.4701	0.3016	0.083*	
C14	0.3271 (5)	0.48905 (15)	0.20032 (13)	0.0655 (5)	
H14	0.2047	0.5340	0.2116	0.079*	

### 2,3-Bis(thiophen-2-yl)pyrido[2,3-b]pyrazine (1) Atomic displacement parameters (Å^2^)

**Table:**

	*U*^11^	*U*^22^	*U*^33^	*U*^12^	*U*^13^	*U*^23^
S1	0.0634 (3)	0.0423 (2)	0.0634 (3)	−0.00914 (17)	0.0164 (2)	−0.01125 (18)
S2	0.0567 (3)	0.1255 (5)	0.0382 (2)	0.0178 (3)	−0.00148 (19)	−0.0182 (3)
N3	0.0466 (6)	0.0332 (5)	0.0375 (6)	0.0006 (4)	0.0055 (5)	−0.0017 (4)
N1	0.0509 (6)	0.0359 (6)	0.0356 (6)	−0.0007 (5)	0.0059 (5)	−0.0035 (4)
C1	0.0410 (6)	0.0336 (6)	0.0327 (6)	0.0021 (5)	0.0022 (5)	0.0003 (5)
C7	0.0407 (6)	0.0335 (6)	0.0327 (6)	0.0022 (5)	0.0018 (5)	−0.0016 (5)
C8	0.0419 (6)	0.0331 (6)	0.0365 (6)	0.0003 (5)	0.0031 (5)	0.0011 (5)
C12	0.0448 (7)	0.0396 (7)	0.0363 (6)	−0.0041 (5)	0.0049 (5)	−0.0068 (5)
C2	0.0473 (7)	0.0361 (6)	0.0329 (6)	0.0031 (5)	0.0053 (5)	−0.0010 (5)
C6	0.0455 (7)	0.0352 (6)	0.0337 (6)	0.0023 (5)	0.0046 (5)	0.0013 (5)
N2	0.0690 (9)	0.0478 (7)	0.0437 (7)	−0.0010 (6)	0.0165 (6)	−0.0107 (6)
C11	0.0544 (9)	0.0437 (8)	0.0639 (11)	−0.0095 (7)	0.0064 (8)	0.0063 (7)
C9	0.0480 (7)	0.0358 (6)	0.0487 (8)	−0.0013 (6)	0.0157 (6)	0.0026 (6)
C10	0.0527 (9)	0.0508 (9)	0.0544 (9)	−0.0041 (7)	0.0159 (7)	0.0061 (7)
C5	0.0544 (9)	0.0436 (8)	0.0474 (8)	−0.0045 (6)	0.0103 (7)	0.0025 (6)
C4	0.0563 (9)	0.0572 (10)	0.0474 (8)	−0.0011 (7)	0.0173 (7)	0.0046 (7)
C3	0.0684 (11)	0.0590 (10)	0.0439 (8)	0.0037 (8)	0.0201 (8)	−0.0072 (7)
C13	0.0728 (11)	0.0462 (8)	0.0495 (9)	0.0139 (8)	0.0069 (8)	−0.0080 (7)
C15	0.0678 (11)	0.0904 (15)	0.0505 (10)	−0.0221 (11)	0.0229 (9)	−0.0331 (10)
C14	0.0807 (13)	0.0528 (10)	0.0645 (12)	0.0034 (9)	0.0221 (10)	−0.0199 (9)

### 2,3-Bis(thiophen-2-yl)pyrido[2,3-b]pyrazine (1) Geometric parameters (Å, º)

**Table:**

S1—C8	1.7229 (14)	N2—C3	1.314 (2)
S1—C11	1.686 (2)	C11—H11	0.9300
S2—C12	1.7060 (15)	C11—C10	1.343 (3)
S2—C15	1.716 (2)	C9—H9	0.9300
N3—C7	1.3131 (18)	C9—C10	1.422 (2)
N3—C6	1.3613 (18)	C10—H10	0.9300
N1—C1	1.3236 (18)	C5—H5	0.9300
N1—C2	1.3486 (19)	C5—C4	1.356 (2)
C1—C7	1.4452 (19)	C4—H4	0.9300
C1—C8	1.466 (2)	C4—C3	1.402 (3)
C7—C12	1.4852 (19)	C3—H3	0.9300
C8—C9	1.401 (2)	C13—H13	0.9300
C12—C13	1.358 (2)	C13—C14	1.422 (3)
C2—C6	1.413 (2)	C15—H15	0.9300
C2—N2	1.3637 (19)	C15—C14	1.322 (4)
C6—C5	1.409 (2)	C14—H14	0.9300
			
C11—S1—C8	92.41 (8)	C10—C11—H11	123.7
C12—S2—C15	91.49 (10)	C8—C9—H9	124.5
C7—N3—C6	117.71 (12)	C8—C9—C10	111.02 (14)
C1—N1—C2	118.29 (12)	C10—C9—H9	124.5
N1—C1—C7	120.52 (12)	C11—C10—C9	113.49 (16)
N1—C1—C8	115.38 (12)	C11—C10—H10	123.3
C7—C1—C8	124.10 (12)	C9—C10—H10	123.3
N3—C7—C1	121.59 (12)	C6—C5—H5	121.0
N3—C7—C12	115.14 (12)	C4—C5—C6	118.02 (15)
C1—C7—C12	123.26 (12)	C4—C5—H5	121.0
C1—C8—S1	117.68 (10)	C5—C4—H4	120.5
C9—C8—S1	110.41 (11)	C5—C4—C3	119.03 (15)
C9—C8—C1	131.91 (13)	C3—C4—H4	120.5
C7—C12—S2	120.39 (11)	N2—C3—C4	125.32 (15)
C13—C12—S2	111.44 (12)	N2—C3—H3	117.3
C13—C12—C7	128.15 (15)	C4—C3—H3	117.3
N1—C2—C6	120.97 (12)	C12—C13—H13	124.0
N1—C2—N2	117.05 (13)	C12—C13—C14	111.93 (18)
N2—C2—C6	121.98 (14)	C14—C13—H13	124.0
N3—C6—C2	120.89 (13)	S2—C15—H15	124.0
N3—C6—C5	119.97 (13)	C14—C15—S2	111.93 (15)
C5—C6—C2	119.13 (13)	C14—C15—H15	124.0
C3—N2—C2	116.51 (14)	C13—C14—H14	123.4
S1—C11—H11	123.7	C15—C14—C13	113.20 (18)
C10—C11—S1	112.65 (13)	C15—C14—H14	123.4
			
S1—C8—C9—C10	−0.99 (18)	C7—C12—C13—C14	−178.93 (16)
S1—C11—C10—C9	−0.9 (2)	C8—S1—C11—C10	0.26 (16)
S2—C12—C13—C14	−0.7 (2)	C8—C1—C7—N3	−178.50 (13)
S2—C15—C14—C13	−1.6 (3)	C8—C1—C7—C12	2.9 (2)
N3—C7—C12—S2	−92.59 (15)	C8—C9—C10—C11	1.2 (2)
N3—C7—C12—C13	85.5 (2)	C12—S2—C15—C14	1.07 (18)
N3—C6—C5—C4	178.33 (15)	C12—C13—C14—C15	1.5 (3)
N1—C1—C7—N3	1.6 (2)	C2—N1—C1—C7	0.0 (2)
N1—C1—C7—C12	−176.99 (13)	C2—N1—C1—C8	−179.89 (12)
N1—C1—C8—S1	6.41 (17)	C2—C6—C5—C4	−0.4 (2)
N1—C1—C8—C9	−172.69 (15)	C2—N2—C3—C4	−0.6 (3)
N1—C2—C6—N3	1.3 (2)	C6—N3—C7—C1	−1.7 (2)
N1—C2—C6—C5	−179.99 (14)	C6—N3—C7—C12	177.03 (12)
N1—C2—N2—C3	−179.52 (16)	C6—C2—N2—C3	0.7 (2)
C1—N1—C2—C6	−1.4 (2)	C6—C5—C4—C3	0.5 (3)
C1—N1—C2—N2	178.84 (14)	N2—C2—C6—N3	−178.93 (14)
C1—C7—C12—S2	86.08 (16)	N2—C2—C6—C5	−0.3 (2)
C1—C7—C12—C13	−95.8 (2)	C11—S1—C8—C1	−178.84 (12)
C1—C8—C9—C10	178.15 (15)	C11—S1—C8—C9	0.44 (13)
C7—N3—C6—C2	0.3 (2)	C5—C4—C3—N2	0.0 (3)
C7—N3—C6—C5	−178.40 (13)	C15—S2—C12—C7	178.22 (13)
C7—C1—C8—S1	−173.50 (11)	C15—S2—C12—C13	−0.19 (15)
C7—C1—C8—C9	7.4 (2)		

### 7-Bromo-2,3-bis(thiophen-2-yl)pyrido[2,3-b]pyrazine (2) Crystal data

**Table:**

C_15_H_8_BrN_3_S_2_	*D*_x_ = 1.747 Mg m^−^^3^
*M**_r_* = 374.27	Melting point: 445 K
Monoclinic, *P*2_1_/*c*	Mo *K*α radiation, λ = 0.71073 Å
*a* = 5.8336 (2) Å	Cell parameters from 9441 reflections
*b* = 29.4731 (10) Å	θ = 4.5–32.2°
*c* = 8.3160 (3) Å	µ = 3.18 mm^−^^1^
β = 95.466 (3)°	*T* = 293 K
*V* = 1423.30 (9) Å^3^	Plate, yellow
*Z* = 4	0.33 × 0.24 × 0.22 mm
*F*(000) = 744	

### 7-Bromo-2,3-bis(thiophen-2-yl)pyrido[2,3-b]pyrazine (2) Data collection

**Table:**

Rigaku Xcalibur Sapphire3 diffractometer	5255 independent reflections
Radiation source: Enhance (Mo) X-ray Source	4166 reflections with *I* > 2σ(*I*)
Graphite monochromator	*R*_int_ = 0.034
Detector resolution: 16.1790 pixels mm^-1^	θ_max_ = 33.9°, θ_min_ = 4.2°
ω scans	*h* = −8→8
Absorption correction: multi-scan (CrysAlis PRO; Rigaku OD, 2018)	*k* = −45→45
*T*_min_ = 0.455, *T*_max_ = 1.000	*l* = −12→12
34519 measured reflections	

### 7-Bromo-2,3-bis(thiophen-2-yl)pyrido[2,3-b]pyrazine (2) Refinement

**Table:**

Refinement on *F*^2^	Primary atom site location: structure-invariant direct methods
Least-squares matrix: full	Secondary atom site location: difference Fourier map
*R*[*F*^2^ > 2σ(*F*^2^)] = 0.043	Hydrogen site location: inferred from neighbouring sites
*wR*(*F*^2^) = 0.110	H-atom parameters constrained
*S* = 1.08	*w* = 1/[σ^2^(*F*_o_^2^) + (0.0474*P*)^2^ + 0.940*P*] where *P* = (*F*_o_^2^ + 2*F*_c_^2^)/3
5255 reflections	(Δ/σ)_max_ < 0.001
190 parameters	Δρ_max_ = 0.63 e Å^−^^3^
0 restraints	Δρ_min_ = −0.67 e Å^−^^3^

### 7-Bromo-2,3-bis(thiophen-2-yl)pyrido[2,3-b]pyrazine (2) Special details

**Table:**

Geometry. All esds (except the esd in the dihedral angle between two l.s. planes) are estimated using the full covariance matrix. The cell esds are taken into account individually in the estimation of esds in distances, angles and torsion angles; correlations between esds in cell parameters are only used when they are defined by crystal symmetry. An approximate (isotropic) treatment of cell esds is used for estimating esds involving l.s. planes.
Refinement. Refinement of F^2^ against ALL reflections. The weighted R-factor wR and goodness of fit S are based on F^2^, conventional R-factors R are based on F, with F set to zero for negative F^2^. The threshold expression of F^2^ > 2sigma(F^2^) is used only for calculating R-factors(gt) etc. and is not relevant to the choice of reflections for refinement. R-factors based on F^2^ are statistically about twice as large as those based on F, and R- factors based on ALL data will be even larger.

### 7-Bromo-2,3-bis(thiophen-2-yl)pyrido[2,3-b]pyrazine (2) Fractional atomic coordinates and isotropic or equivalent isotropic displacement parameters (Å^2^)

**Table:**

	*x*	*y*	*z*	*U*_iso_*/*U*_eq_	
Br1	0.69446 (4)	0.018840 (9)	0.30276 (3)	0.04319 (9)	
S2	0.62946 (10)	−0.11087 (2)	−0.49251 (7)	0.04132 (14)	
S1	1.54351 (12)	−0.19256 (2)	−0.09942 (10)	0.05248 (18)	
N3	0.8311 (3)	−0.08963 (6)	−0.1758 (2)	0.0312 (3)	
C12	0.8921 (3)	−0.13124 (7)	−0.4089 (3)	0.0294 (4)	
C7	0.9600 (3)	−0.12031 (7)	−0.2402 (2)	0.0290 (4)	
C2	1.1218 (4)	−0.08478 (7)	0.0484 (3)	0.0314 (4)	
C6	0.9043 (3)	−0.07298 (7)	−0.0283 (2)	0.0290 (4)	
C13	1.0082 (4)	−0.15121 (8)	−0.5255 (3)	0.0354 (4)	
H13	1.1549	−0.1635	−0.5056	0.042*	
N1	1.2404 (3)	−0.11997 (7)	−0.0083 (2)	0.0342 (4)	
N2	1.2152 (4)	−0.06423 (8)	0.1847 (3)	0.0428 (5)	
C1	1.1566 (4)	−0.13963 (7)	−0.1436 (3)	0.0298 (4)	
C5	0.7682 (4)	−0.04198 (8)	0.0495 (3)	0.0327 (4)	
H5	0.6198	−0.0346	0.0066	0.039*	
C4	0.8620 (4)	−0.02319 (7)	0.1898 (3)	0.0330 (4)	
C8	1.2653 (4)	−0.18271 (7)	−0.1791 (3)	0.0331 (4)	
C14	0.8825 (4)	−0.15116 (8)	−0.6789 (3)	0.0386 (5)	
H14	0.9366	−0.1637	−0.7707	0.046*	
C15	0.6754 (4)	−0.13092 (8)	−0.6780 (3)	0.0395 (5)	
H15	0.5699	−0.1283	−0.7686	0.047*	
C3	1.0885 (4)	−0.03388 (9)	0.2503 (3)	0.0413 (5)	
H3	1.1521	−0.0186	0.3419	0.050*	
C9	1.1615 (5)	−0.22302 (8)	−0.2534 (3)	0.0445 (6)	
H9	1.0113	−0.2255	−0.3013	0.053*	
C10	1.3303 (6)	−0.25845 (9)	−0.2402 (4)	0.0575 (8)	
H10	1.3004	−0.2874	−0.2817	0.069*	
C11	1.5344 (6)	−0.24684 (10)	−0.1638 (4)	0.0589 (8)	
H11	1.6584	−0.2667	−0.1479	0.071*	

### 7-Bromo-2,3-bis(thiophen-2-yl)pyrido[2,3-b]pyrazine (2) Atomic displacement parameters (Å^2^)

**Table:**

	*U*^11^	*U*^22^	*U*^33^	*U*^12^	*U*^13^	*U*^23^
Br1	0.04705 (15)	0.04449 (14)	0.03805 (13)	0.00908 (10)	0.00417 (10)	−0.01197 (10)
S2	0.0361 (3)	0.0475 (3)	0.0388 (3)	0.0120 (2)	−0.0047 (2)	−0.0046 (2)
S1	0.0458 (3)	0.0422 (3)	0.0694 (5)	0.0123 (3)	0.0051 (3)	0.0048 (3)
N3	0.0301 (8)	0.0319 (9)	0.0308 (8)	0.0037 (7)	−0.0023 (6)	−0.0041 (7)
C12	0.0274 (9)	0.0279 (9)	0.0320 (9)	0.0004 (7)	−0.0016 (7)	−0.0012 (7)
C7	0.0294 (9)	0.0272 (9)	0.0301 (9)	0.0005 (7)	0.0007 (7)	−0.0005 (7)
C2	0.0304 (9)	0.0329 (10)	0.0299 (9)	0.0045 (8)	−0.0019 (7)	−0.0015 (8)
C6	0.0291 (9)	0.0282 (9)	0.0291 (9)	0.0034 (7)	−0.0003 (7)	−0.0006 (7)
C13	0.0326 (10)	0.0393 (11)	0.0337 (10)	0.0016 (9)	0.0010 (8)	−0.0031 (9)
N1	0.0333 (9)	0.0353 (9)	0.0327 (9)	0.0084 (7)	−0.0031 (7)	−0.0012 (7)
N2	0.0382 (10)	0.0502 (12)	0.0376 (10)	0.0112 (9)	−0.0093 (8)	−0.0104 (9)
C1	0.0306 (9)	0.0267 (9)	0.0323 (10)	0.0038 (7)	0.0037 (7)	0.0003 (7)
C5	0.0306 (9)	0.0343 (10)	0.0323 (10)	0.0057 (8)	−0.0012 (8)	−0.0021 (8)
C4	0.0370 (10)	0.0316 (10)	0.0306 (10)	0.0021 (8)	0.0041 (8)	−0.0027 (8)
C8	0.0367 (10)	0.0285 (9)	0.0345 (10)	0.0057 (8)	0.0058 (8)	0.0030 (8)
C14	0.0479 (13)	0.0365 (11)	0.0313 (10)	−0.0003 (9)	0.0031 (9)	−0.0030 (8)
C15	0.0481 (13)	0.0368 (11)	0.0314 (11)	0.0015 (10)	−0.0085 (9)	−0.0011 (8)
C3	0.0432 (12)	0.0451 (13)	0.0334 (11)	0.0070 (10)	−0.0076 (9)	−0.0106 (9)
C9	0.0523 (14)	0.0343 (12)	0.0476 (13)	0.0153 (10)	0.0085 (11)	0.0000 (10)
C10	0.075 (2)	0.0290 (12)	0.072 (2)	0.0045 (12)	0.0257 (16)	−0.0035 (12)
C11	0.0594 (18)	0.0402 (14)	0.080 (2)	0.0221 (13)	0.0240 (16)	0.0075 (14)

### 7-Bromo-2,3-bis(thiophen-2-yl)pyrido[2,3-b]pyrazine (2) Geometric parameters (Å, º)

**Table:**

Br1—C4	1.884 (2)	N1—C1	1.318 (3)
S2—C12	1.729 (2)	N2—C3	1.312 (3)
S2—C15	1.697 (2)	C1—C8	1.462 (3)
S1—C8	1.718 (2)	C5—H5	0.9300
S1—C11	1.686 (3)	C5—C4	1.358 (3)
N3—C7	1.321 (3)	C4—C3	1.404 (3)
N3—C6	1.352 (3)	C8—C9	1.446 (4)
C12—C7	1.457 (3)	C14—H14	0.9300
C12—C13	1.368 (3)	C14—C15	1.348 (3)
C7—C1	1.452 (3)	C15—H15	0.9300
C2—C6	1.408 (3)	C3—H3	0.9300
C2—N1	1.356 (3)	C9—H9	0.9300
C2—N2	1.353 (3)	C9—C10	1.432 (4)
C6—C5	1.408 (3)	C10—H10	0.9300
C13—H13	0.9300	C10—C11	1.339 (5)
C13—C14	1.410 (3)	C11—H11	0.9300
			
C15—S2—C12	91.95 (11)	C4—C5—H5	121.3
C11—S1—C8	92.17 (14)	C5—C4—Br1	120.71 (17)
C7—N3—C6	118.26 (18)	C5—C4—C3	120.3 (2)
C7—C12—S2	117.37 (15)	C3—C4—Br1	118.93 (17)
C13—C12—S2	110.13 (16)	C1—C8—S1	118.76 (17)
C13—C12—C7	132.08 (19)	C9—C8—S1	111.58 (17)
N3—C7—C12	115.37 (18)	C9—C8—C1	128.9 (2)
N3—C7—C1	119.60 (18)	C13—C14—H14	123.7
C1—C7—C12	125.02 (18)	C15—C14—C13	112.6 (2)
N1—C2—C6	119.96 (19)	C15—C14—H14	123.7
N2—C2—C6	122.8 (2)	S2—C15—H15	123.9
N2—C2—N1	117.08 (19)	C14—C15—S2	112.24 (17)
N3—C6—C2	120.93 (19)	C14—C15—H15	123.9
N3—C6—C5	120.64 (18)	N2—C3—C4	123.5 (2)
C5—C6—C2	118.38 (19)	N2—C3—H3	118.3
C12—C13—H13	123.5	C4—C3—H3	118.3
C12—C13—C14	113.0 (2)	C8—C9—H9	126.0
C14—C13—H13	123.5	C10—C9—C8	108.0 (3)
C1—N1—C2	118.22 (18)	C10—C9—H9	126.0
C3—N2—C2	117.1 (2)	C9—C10—H10	122.5
C7—C1—C8	124.32 (19)	C11—C10—C9	115.1 (3)
N1—C1—C7	120.60 (18)	C11—C10—H10	122.5
N1—C1—C8	114.94 (19)	S1—C11—H11	123.4
C6—C5—H5	121.3	C10—C11—S1	113.1 (2)
C4—C5—C6	117.4 (2)	C10—C11—H11	123.4
			
Br1—C4—C3—N2	176.7 (2)	C6—C2—N2—C3	3.8 (4)
S2—C12—C7—N3	−10.8 (3)	C6—C5—C4—Br1	179.77 (16)
S2—C12—C7—C1	170.70 (17)	C6—C5—C4—C3	1.6 (3)
S2—C12—C13—C14	−1.8 (3)	C13—C12—C7—N3	161.0 (2)
S1—C8—C9—C10	−1.7 (3)	C13—C12—C7—C1	−17.6 (4)
N3—C7—C1—N1	−16.0 (3)	C13—C14—C15—S2	0.8 (3)
N3—C7—C1—C8	159.4 (2)	N1—C2—C6—N3	−13.7 (3)
N3—C6—C5—C4	−173.5 (2)	N1—C2—C6—C5	168.7 (2)
C12—S2—C15—C14	−1.6 (2)	N1—C2—N2—C3	−172.1 (2)
C12—C7—C1—N1	162.4 (2)	N1—C1—C8—S1	−25.5 (3)
C12—C7—C1—C8	−22.2 (3)	N1—C1—C8—C9	143.6 (2)
C12—C13—C14—C15	0.7 (3)	N2—C2—C6—N3	170.5 (2)
C7—N3—C6—C2	5.8 (3)	N2—C2—C6—C5	−7.0 (4)
C7—N3—C6—C5	−176.7 (2)	N2—C2—N1—C1	−177.9 (2)
C7—C12—C13—C14	−174.0 (2)	C1—C8—C9—C10	−171.4 (2)
C7—C1—C8—S1	158.88 (17)	C5—C4—C3—N2	−5.1 (4)
C7—C1—C8—C9	−32.0 (4)	C8—S1—C11—C10	−1.2 (3)
C2—C6—C5—C4	4.0 (3)	C8—C9—C10—C11	0.9 (4)
C2—N1—C1—C7	8.1 (3)	C15—S2—C12—C7	175.40 (18)
C2—N1—C1—C8	−167.7 (2)	C15—S2—C12—C13	1.91 (19)
C2—N2—C3—C4	2.3 (4)	C9—C10—C11—S1	0.3 (4)
C6—N3—C7—C12	−170.23 (19)	C11—S1—C8—C1	172.6 (2)
C6—N3—C7—C1	8.4 (3)	C11—S1—C8—C9	1.7 (2)
C6—C2—N1—C1	6.1 (3)		

### 7-Bromo-2,3-bis(thiophen-2-yl)pyrido[2,3-b]pyrazine (2) Hydrogen-bond geometry (Å, º)

**Table:**

*D*—H···*A*	*D*—H	H···*A*	*D*···*A*	*D*—H···*A*
C3—H3···Br1^i^	0.93	3.01	3.836 (1)	150
C5—H5···Br1^ii^	0.93	3.05	3.851 (1)	145
C15—H15···N1^iii^	0.93	2.65	3.572 (1)	175
C11—H11···C14^iv^	0.93	2.78	3.637 (1)	155

Symmetry codes: (i) −*x*+2, −*y*, −*z*+1; (ii) −*x*+1, −*y*, −*z*; (iii) *x*−1, *y*, *z*−1; (iv) *x*+1, −*y*−1/2, *z*+1/2.

## References

[bb1] Bi, W.-Y., Chai, W.-L., Lu, X.-Q., Song, J.-R. & Bao, F. (2009). *J. Coord. Chem.* **62**, 1928–1938.

[bb2] Crundwell, G. (2003). *J. Chem. Crystallogr.* **33**, 239–244.

[bb3] Crundwell, G. (2013). *Acta Cryst.* E**69**, m164. 10.1107/S1600536813004510 PMC3588418 23476506

[bb4] Crundwell, G., Cantalupo, S., D. C. Foss, P., McBurney, B., Kopp, K., L. Westcott, B., Updegraff III, J., Zeller, M. & D. Hunter, A. (2014). *Open J. Inorg. Chem.* **04**, 10–17.

[bb5] Dolomanov, O. V., Bourhis, L. J., Gildea, R. J., Howard, J. A. K. & Puschmann, H. (2009). *J. Appl. Cryst.* **42**, 339–341.

[bb6] Farrugia, L. J. (2012). *J. Appl. Cryst.* **45**, 849–854.

[bb7] Ghosh, T., Maiya, B. G. & Samanta, A. (2006). *Dalton Trans.* pp. 795–801. 10.1039/b510469f 16437174

[bb8] Groom, C. R., Bruno, I. J., Lightfoot, M. P. & Ward, S. C. (2016). *Acta Cryst.* B**72**, 171–179. 10.1107/S2052520616003954 PMC4822653 27048719

[bb9] Gupta, S. & Milton, M. D. (2018). *New J. Chem.* **42**, 2838–2849.

[bb10] Kekesi, L., Dancso, A., Illyes, E., Boros, S., Pato, J., Greff, Z., Nemeth, G., Garamvolgyi, R., Baska, F., Orfi, L. & Keri, G. (2014). *Lett. Org. Chem.* **11**, 651–656.

[bb11] Koch, P., Schollmeyer, D. & Laufer, S. (2009*a*). *Acta Cryst.* E**65**, o2512. 10.1107/S1600536809037295 PMC2970199 21577959

[bb12] Koch, P., Schollmeyer, D. & Laufer, S. (2009*b*). *Acta Cryst.* E**65**, o2546. 10.1107/S1600536809037970 PMC2970414 21577988

[bb13] Koch, P., Schollmeyer, D. & Laufer, S. (2009*c*). *Acta Cryst.* E**65**, o2557. 10.1107/S1600536809038173 PMC2970374 21577998

[bb14] Lassagne, F., Chevallier, F., Roisnel, T., Dorcet, V., Mongin, F. & Domingo, L. R. (2015). *Synthesis*, **47**, 2680–2689.

[bb15] Macrae, C. F., Bruno, I. J., Chisholm, J. A., Edgington, P. R., McCabe, P., Pidcock, E., Rodriguez-Monge, L., Taylor, R., van de Streek, J. & Wood, P. A. (2008). *J. Appl. Cryst.* **41**, 466–470.

[bb16] Manivannan, R., Satheshkumar, A., El-Mossalamy, E. H., Al-Harbi, L. M., Kosa, S. A. & Elango, K. P. (2015). *New J. Chem.* **39**, 3936–3947.

[bb17] Rigaku OD (2018). *CrysAlis PRO*. Rigaku Oxford Diffraction, Yarnton, England.

[bb18] Shanmuga Sundara Raj, S., Fun, H.-K., Chen, X.-F., Zhu, X.-H. & You, X.-Z. (1999). *Acta Cryst.* C**55**, 2035–2037.

[bb19] Sheldrick, G. M. (2008). *Acta Cryst.* A**64**, 112–122. 10.1107/S0108767307043930 18156677

[bb20] Xu, L., Zhao, Y., Long, G., Wang, Y., Zhao, J., Li, D., Li, J., Ganguly, R., Li, Y., Sun, H., Sun, X. W. & Zhang, Q. (2015). *RSC Adv.* **5**, 63080–63086.

[bb21] Yeo, B. R., Hallett, A. J., Kariuki, B. M. & Pope, S. J. A. (2010). *Polyhedron*, **29**, 1088–1094.

