# Supplementary material for: Crystal structures of 2,3-bis­(thio­phen-2-yl)pyrido[2,3-b]pyrazine and 7-bromo-2,3-bis­(thio­phen-2-yl)pyrido[2,3-b]pyrazine
Source: Acta Crystallogr E Crystallogr Commun. 2019 Jan 1;75(Pt 1):89–93. doi: 10.1107/S2056989018016882 (PMC6323875; doi:10.1107/S2056989018016882)
Supplement: Supplementary file 6 [file e-75-00089-sup6.pdf]

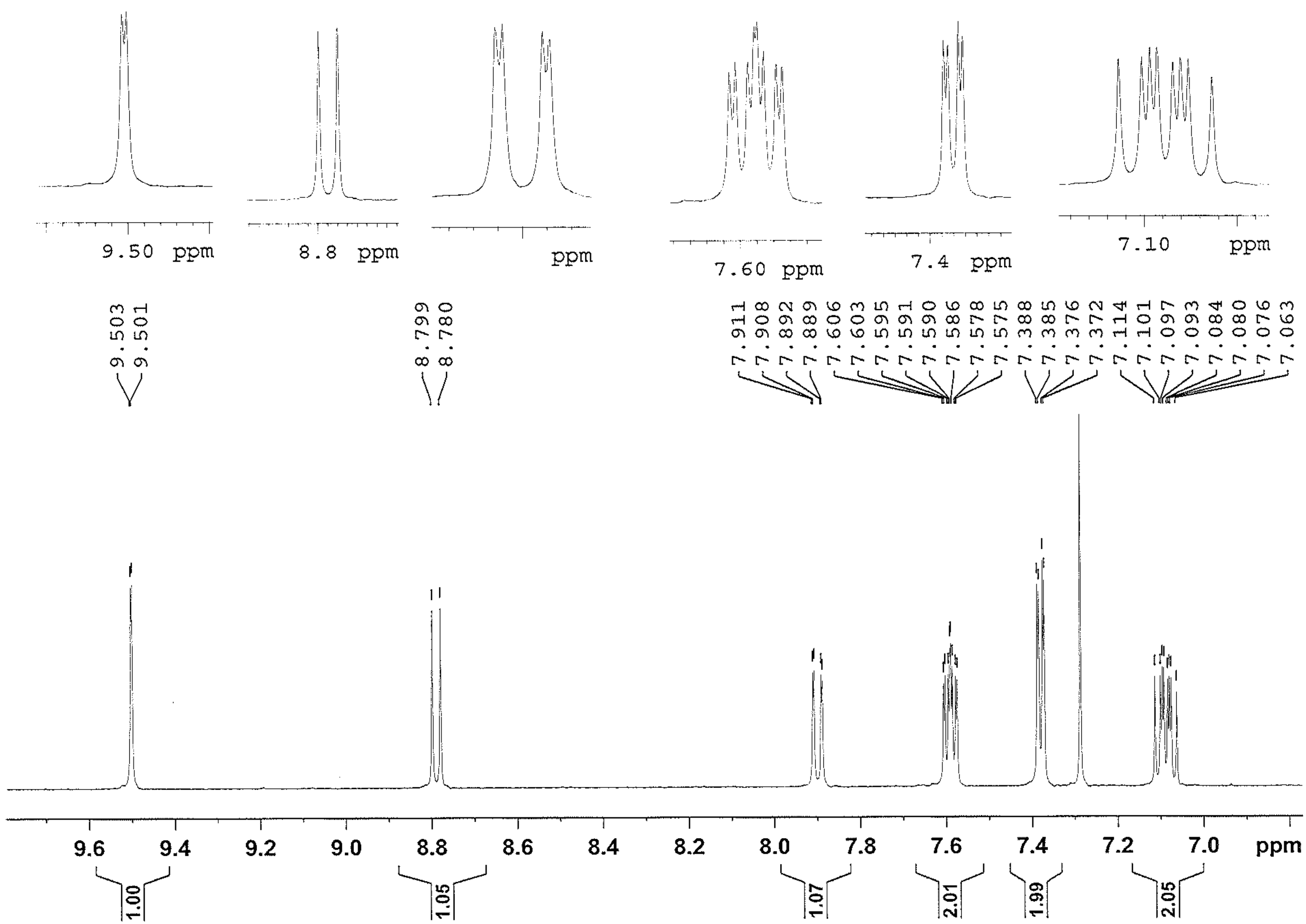
9.50 ppm
8.8 ppm
ppm
7.60 ppm
7.4 ppm
7.10 ppm
9.503
9.501
8.799
8.780
7.911
7.908
7.892
7.889
7.606
7.603
7.595
7.591
7.590
7.586
7.578
7.575
7.388
7.385
7.376
7.372
7.114
7.101
7.097
7.093
7.084
7.080
7.076
7.063
9.6 9.4 9.2 9.0 8.8 8.6 8.4 8.2 8.0 7.8 7.6 7.4 7.2 7.0 ppm
1.00
1.05
1.07
2.01
1.99
2.05

I

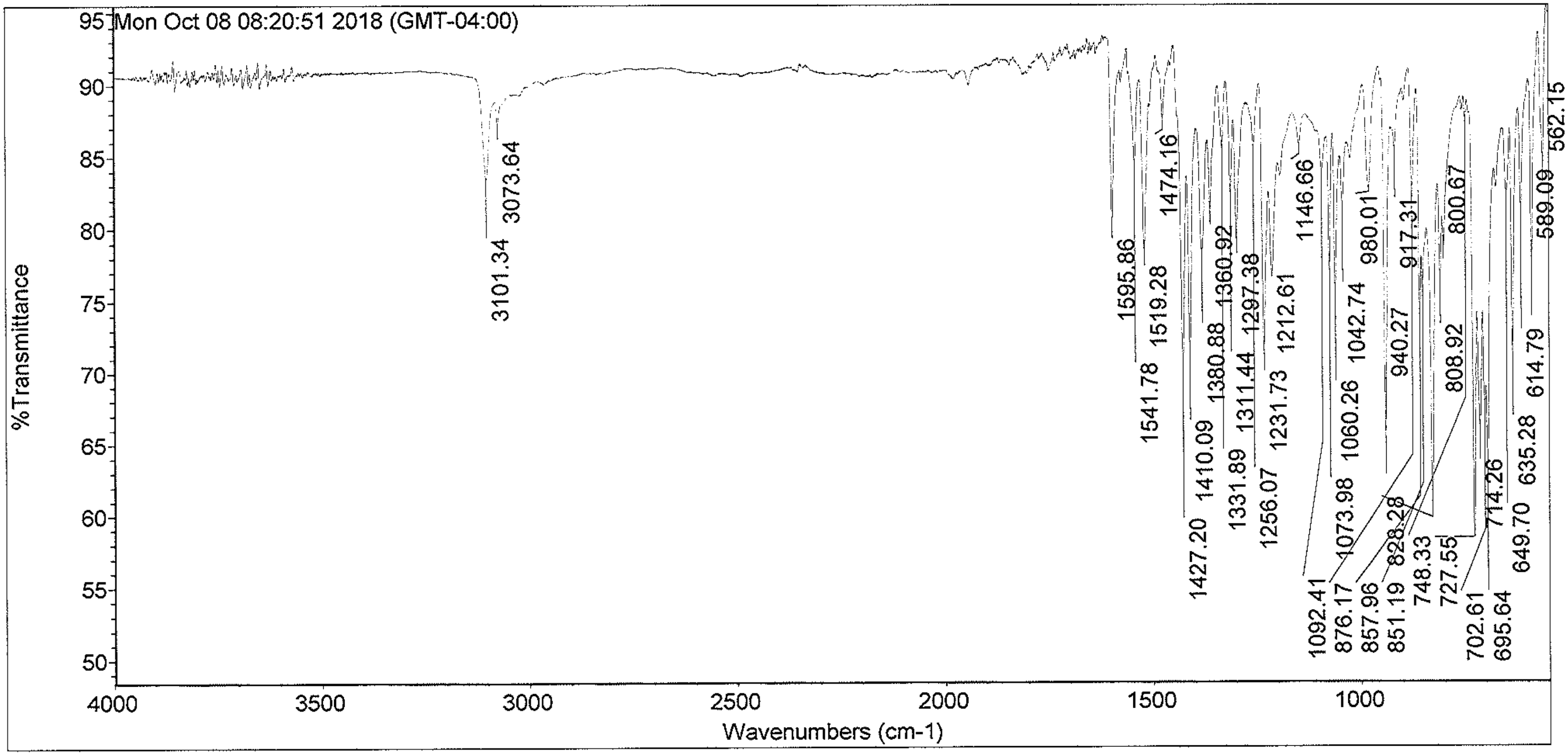

Mon Oct 08 08:24:37 2018 (GMT-04:00)
FIND PEAKS:
Spectrum: Mon Oct 08 08:20:51 2018 (GMT-04:00)
Region: 4000.12 550.10
Absolute threshold: 89.082
Sensitivity: 50
Peak list:

Position:	562.15	Intensity:	83.782
Position:	589.09	Intensity:	75.950
Position:	614.79	Intensity:	81.145
Position:	635.28	Intensity:	71.292
Position:	649.70	Intensity:	82.479
Position:	695.64	Intensity:	63.689
Position:	702.61	Intensity:	67.334
Position:	714.26	Intensity:	66.183

I

Position:	727.55	Intensity:	58.389
Position:	748.33	Intensity:	87.506
Position:	800.67	Intensity:	78.461
Position:	808.92	Intensity:	76.632
Position:	828.28	Intensity:	60.374
Position:	851.19	Intensity:	76.043
Position:	857.96	Intensity:	73.387
Position:	876.17	Intensity:	81.979
Position:	917.31	Intensity:	85.485
Position:	940.27	Intensity:	65.471
Position:	980.01	Intensity:	82.703
Position:	1042.74	Intensity:	81.583
Position:	1060.26	Intensity:	75.297
Position:	1073.98	Intensity:	77.082
Position:	1092.41	Intensity:	83.894
Position:	1146.66	Intensity:	85.796
Position:	1212.61	Intensity:	76.748
Position:	1231.73	Intensity:	71.340
Position:	1256.07	Intensity:	85.580
Position:	1297.38	Intensity:	79.200
Position:	1311.44	Intensity:	82.056
Position:	1331.89	Intensity:	85.781
Position:	1360.92	Intensity:	81.198
Position:	1380.88	Intensity:	78.186
Position:	1410.09	Intensity:	71.322
Position:	1427.20	Intensity:	70.750
Position:	1474.16	Intensity:	87.468
Position:	1519.28	Intensity:	78.348
Position:	1541.78	Intensity:	84.590
Position:	1595.86	Intensity:	80.191
Position:	3073.64	Intensity:	87.231
Position:	3101.34	Intensity:	83.448

I

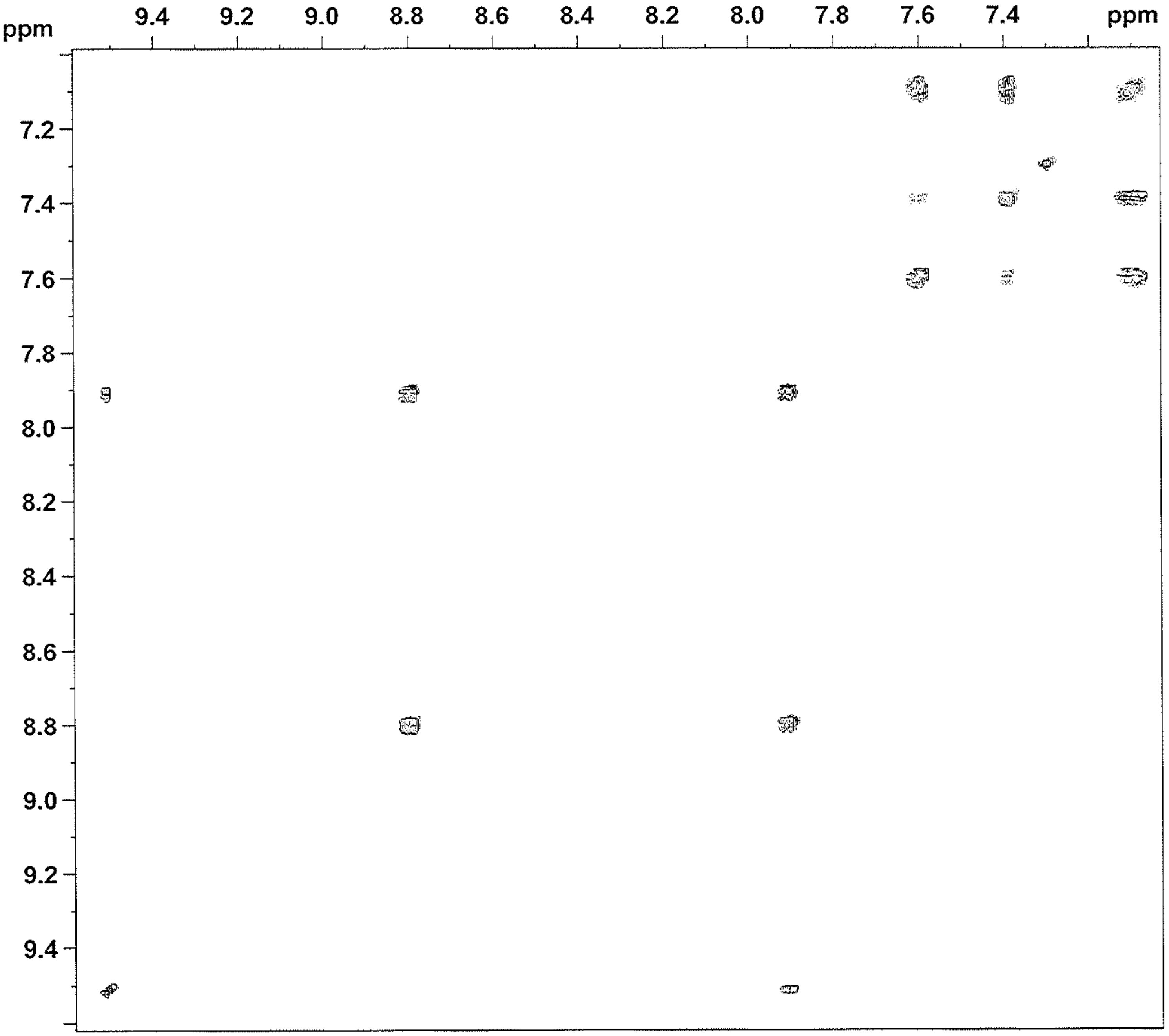
ppm
9.4
9.2
9.0
8.8
8.6
8.4
8.2
8.0
7.8
7.6
7.4
ppm
7.2
7.4
7.6
7.8
8.0
8.2
8.4
8.6
8.8
9.0
9.2
9.4

II Br

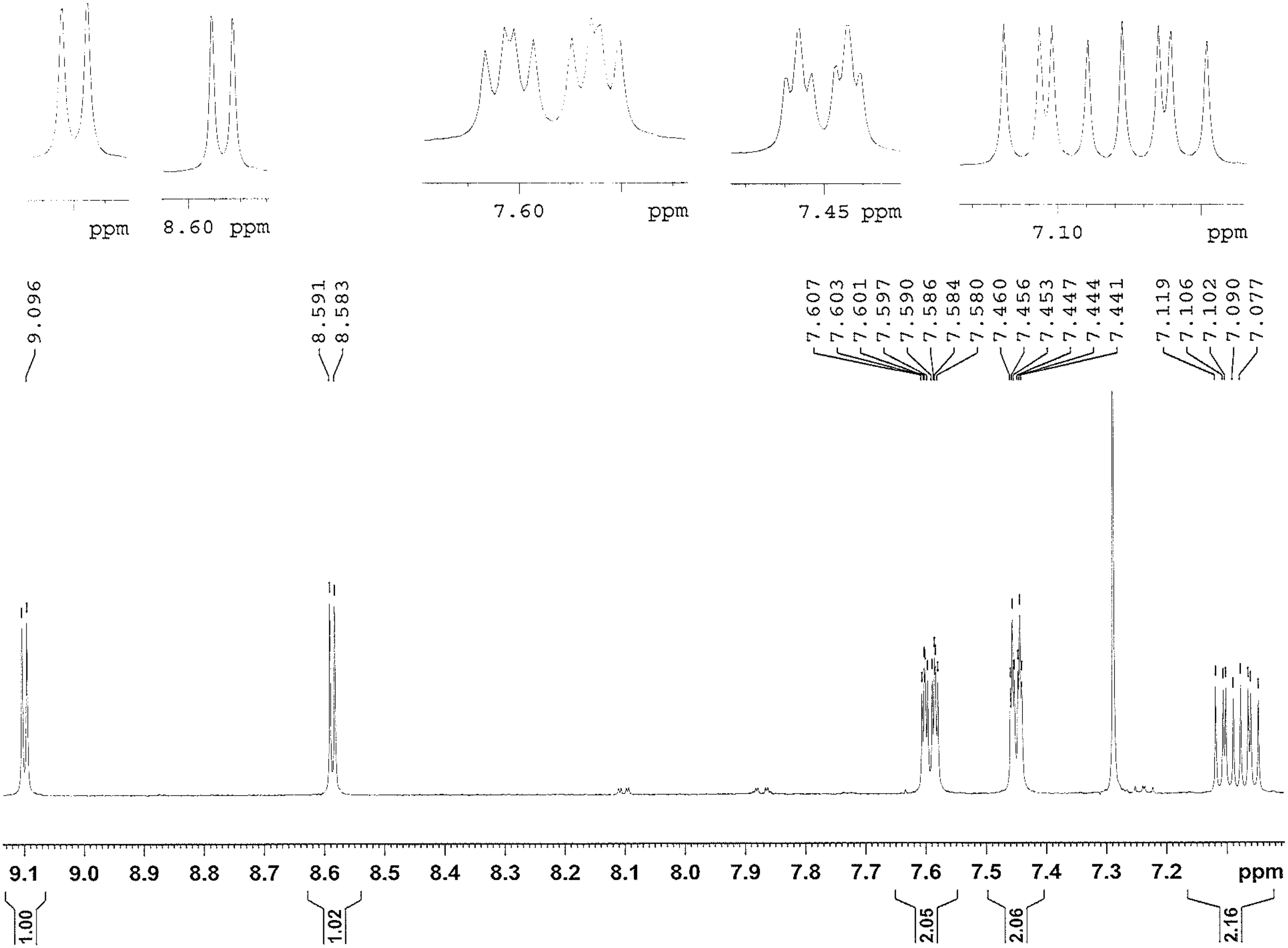

ppm
8.60 ppm
7.60
ppm
7.45 ppm
7.10
ppm
9.096
8.591
8.583
7.607
7.603
7.601
7.597
7.590
7.586
7.584
7.580
7.460
7.456
7.453
7.447
7.444
7.441
7.119
7.106
7.102
7.090
7.077
9.1 9.0 8.9 8.8 8.7 8.6 8.5 8.4 8.3 8.2 8.1 8.0 7.9 7.8 7.7 7.6 7.5 7.4 7.3 7.2 ppm
1.00
1.02
2.05
2.06
2.16

II Br

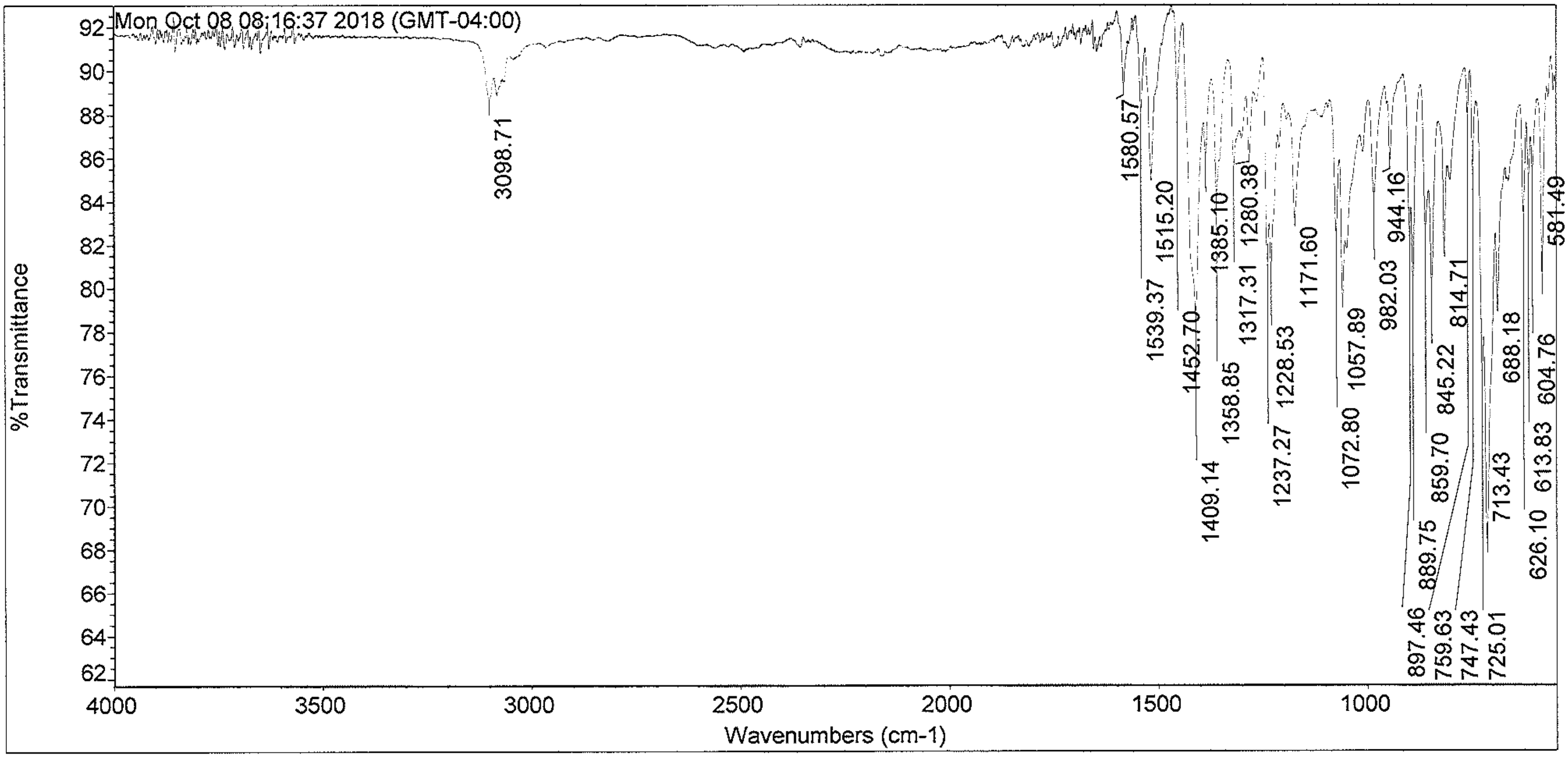

Mon Oct 08 08:17:18 2018 (GMT-04:00)
FIND PEAKS:

Spectrum:	Mon Oct 08 08:16:37 2018 (GMT-04:00)		
Region:	4000.12	550.10	
Absolute threshold:	89.870		
Sensitivity:	50		
Peak list:			

Position:	581.49	Intensity:	80.576
Position:	604.76	Intensity:	85.340
Position:	613.83	Intensity:	85.054
Position:	626.10	Intensity:	83.354
Position:	688.18	Intensity:	79.167
Position:	713.43	Intensity:	68.323
Position:	725.01	Intensity:	76.022
Position:	747.43	Intensity:	85.586

II

Position:	759.63	Intensity:	87.954
Position:	814.71	Intensity:	81.721
Position:	845.22	Intensity:	80.162
Position:	859.70	Intensity:	82.748
Position:	889.75	Intensity:	80.013
Position:	897.46	Intensity:	82.400
Position:	944.16	Intensity:	85.778
Position:	982.03	Intensity:	84.304
Position:	1057.89	Intensity:	79.385
Position:	1072.80	Intensity:	82.501
Position:	1171.60	Intensity:	83.244
Position:	1228.53	Intensity:	80.830
Position:	1237.27	Intensity:	81.092
Position:	1280.38	Intensity:	86.037
Position:	1317.31	Intensity:	85.493
Position:	1358.85	Intensity:	84.343
Position:	1385.10	Intensity:	85.965
Position:	1409.14	Intensity:	78.862
Position:	1452.70	Intensity:	89.361
Position:	1515.20	Intensity:	85.162
Position:	1539.37	Intensity:	88.061
Position:	1580.57	Intensity:	89.240
Position:	3098.71	Intensity:	88.678

II

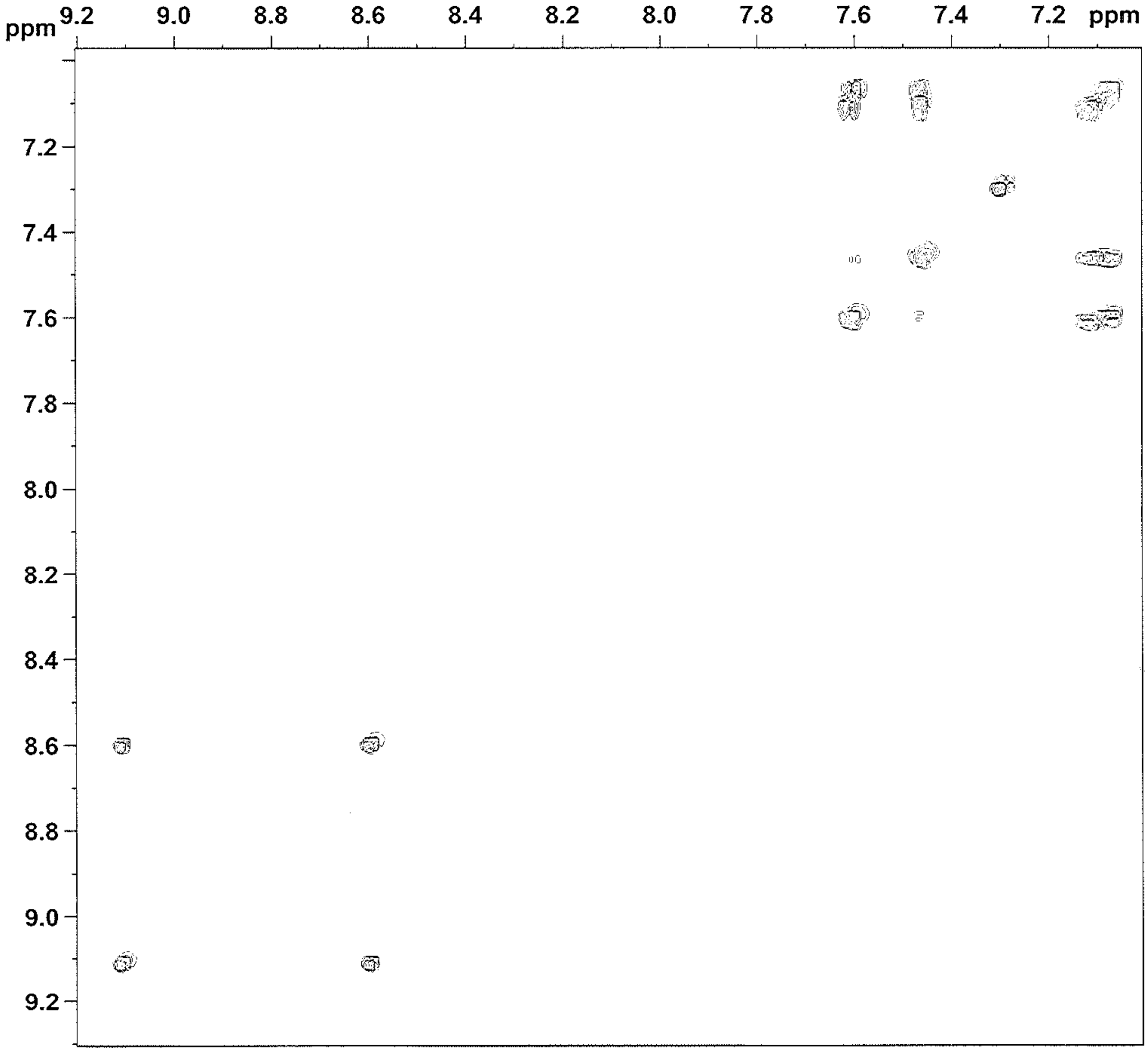
ppm
9.2
9.0
8.8
8.6
8.4
8.2
8.0
7.8
7.6
7.4
7.2
ppm
7.2
7.4
7.6
7.8
8.0
8.2
8.4
8.6
8.8
9.0
9.2
